# Augmented Reality-Guided Apicoectomy Based on Maxillofacial CBCT Scans

**DOI:** 10.3390/diagnostics13193037

**Published:** 2023-09-25

**Authors:** Bernhard Remschmidt, Marcus Rieder, Christina Gsaxner, Jan Gaessler, Michael Payer, Juergen Wallner

**Affiliations:** 1 Division of Oral and Maxillofacial Surgery, Department of Dental Medicine and Oral Health, Medical University of Graz, 8036 Graz, Austria; 2 Division of Oral Surgery and Orthodontics, Department of Dental Medicine and Oral Health, Medical University of Graz, 8010 Graz, Austria; 3 Institute of Computer Graphics and Vision, Graz University of Technology, 8010 Graz, Austria

**Keywords:** apicoectomy, endodontic surgery, root-end resection, surgery, computer-assisted, augmented reality, cone beam computed tomography

## Abstract

Implementation of augmented reality (AR) image guidance systems using preoperative cone beam computed tomography (CBCT) scans in apicoectomies promises to help surgeons overcome iatrogenic complications associated with this procedure. This study aims to evaluate the intraoperative feasibility and usability of HoloLens 2, an established AR image guidance device, in the context of apicoectomies. Three experienced surgeons carried out four AR-guided apicoectomies each on human cadaver head specimens. Preparation and operating times of each procedure, as well as the subjective usability of HoloLens for AR image guidance in apicoectomies using the System Usability Scale (SUS), were measured. In total, twelve AR-guided apicoectomies on six human cadaver head specimens were performed (*n* = 12). The average preparation time amounted to 162 (±34) s. The surgical procedure itself took on average 9 (±2) min. There was no statistically significant difference between the three surgeons. Quantification of the usability of HoloLens revealed a mean SUS score of 80.4 (±6.8), indicating an “excellent” usability level. In conclusion, this study implies the suitability, practicality, and simplicity of AR image guidance systems such as the HoloLens in apicoectomies and advocates their routine implementation.

## 1. Introduction

Cone beam computed tomography (CBCT) was developed by Arai and colleagues in 1997 with the intention of providing a more compact modification of computed tomography (CT) scans, specifically to be used in dentistry [1]. Since then, CBCT has become an indispensable aid for diagnostics and treatment planning [2]. In addition to other indications, endodontics represent one of the main areas of clinical CBCT application [1]. Regarding the detection of periapical lesions, CBCT imaging has proven to have higher sensitivity and specificity in comparison with conventional periapical or panoramic radiographs [3]. In addition to its diagnostic value, CBCT has been incorporated in the preoperative planning of apicoectomies [4]. Using microsurgical techniques, apicoectomies have a high success rate [5,6]. Yet, there are complications that can lead to failure. Considering surgeon-dependent factors, the resection of the false root end or an injury to adjacent structures are among the most frequent complications [7]. Aiming to help surgeons avoid these complications, several computer-based solutions (e.g., intraoperative navigation systems) have been tested and introduced in the recent past [8,9,10]. Nevertheless, conventional image guidance has its limitations. Primarily, the surgeon has to divide their attention by focusing on a monitor and the patient simultaneously, which increases mental workload and deteriorates hand-eye coordination [11,12]. Secondly, complex three-dimensional (3D) data, such as CBCT scans, need to be projected onto monitors in two dimensions separated from the patient by a computer workstation. Thirdly, conventionally available image guidance systems are highly expensive, industry-driven, license protected- and usually involve complex and bulky setups. In dentistry, the additional expense and effort involved in assembling a guidance system is often considered unjustified for the performed procedure [13,14].

Augmented reality (AR) has the potential to overcome these limitations. AR is a transformative technology that expands the users’ understanding and awareness of their environment through the incorporation of virtual content into reality [15,16]. In the medical sector, AR offers the possibility of merging imaging data with the patient, thus enabling new dimensions of 3D visualizations. AR fundamentally diverges from virtual reality (VR) in its approach, as AR preserves the user’s perceptual connection to the real world while overlaying virtual elements, whereas VR immerses the user in an entirely synthetic virtual environment, severing their sensory ties to the real world [17]. In the medical sector, AR offers the possibility of merging imaging data with the patient, thus enabling new dimensions of 3D visualizations. New perspectives for pedagogical and didactic methods arise, and real-time navigation guidance is thus made possible. Specializations in dentistry that utilize this new technology include the orthognathic section of oral surgery, oral surgery and implantology, orthodontics, and endodontics. The use of AR in these fields takes advantage of AR’s ability to superimpose structures needing protection as well as target structures into the user’s field of view [18,19,20,21,22,23,24]. The leading innovative AR device that has garnered significant attention in the healthcare domain is the HoloLens (HL) (Microsoft Corp., Redmond, WA, USA) [25].

The HL is a see-through head-mounted AR display that enables users to perceive and manipulate virtual objects within their physical environment. Compared with conventional image guidance systems, the HL circumvents the surgeons’ need to branch their attention between monitor and patient, providing real 3D visualization of patient-specific image data with a stereoscopic display. It is also comparably cheap and has a very slim form factor. That said, the application of the HL in dentistry has been limited up to now. Despite the proposition of several systems for 3D visualization of dental data [26,27,28], the majority of these systems lack comprehensive guidance capabilities. However, the proof-of-concept HL-based dental guidance systems described by Pellegrino et al. and Song et al. still rely on external navigation systems and invasive markers, respectively [18,29]. Consequently, it is evident that the true potential of see-through AR in dentistry remains largely unexplored to date.

In the context of dental procedures involving the craniofacial region, there has been a notable development in the form of a practical image guidance system based on the HL. This system, proposed by Gsaxner et al., provides several advantages, including simplified assembly, and eliminates the need for external hardware or infrastructure [30,31,32]. In fact, the system is based on routinely acquired pre-interventional CT scans. From the CT scan, data about a patient’s skin surface can be extracted. Then, the AR system achieves an automatic and markerless registration between the extracted skin model and the physical patient, solely by using the adeptness of the HL hardware. 

The current study aims to evaluate the potential benefits of incorporating such an AR guidance system using dental CBCT scans in apicoectomy procedures.

## 2. Materials and Methods

Three experienced surgeons carried out four guided apicoectomies each on human cadaver head specimens to evaluate the potential benefits of an HL-based AR guidance system. Each specimen underwent a prior CBCT scan. The maxillary lateral incisor teeth, as well as the entire facial skeleton and skin surface, were segmented for visualization in AR. These data were precisely overlaid with the specimen before the surgery.

### 2.1. Data and Specimen Collection

The current study was carried out using tissues of human cadaver head specimens provided by the Division of Macroscopic and Clinical Anatomy at the Medical University of Graz. All cadaver specimens were preserved using Thiel’s method, a high-quality embalming technique used for advanced surgical training and anatomical research purposes [33,34]. Protected by the Styrian Death and Funeral Act of 2010, permission to use post-mortem tissues was granted after undergoing an institutional review. All specimens were handled in conformity with the strict rules of the donation program of the aforementioned division. Furthermore, approval of the protocol was obtained from the institutional ethics committee (IRB00002556, re: 31-416 ex 18/19).

**Figure 1:**
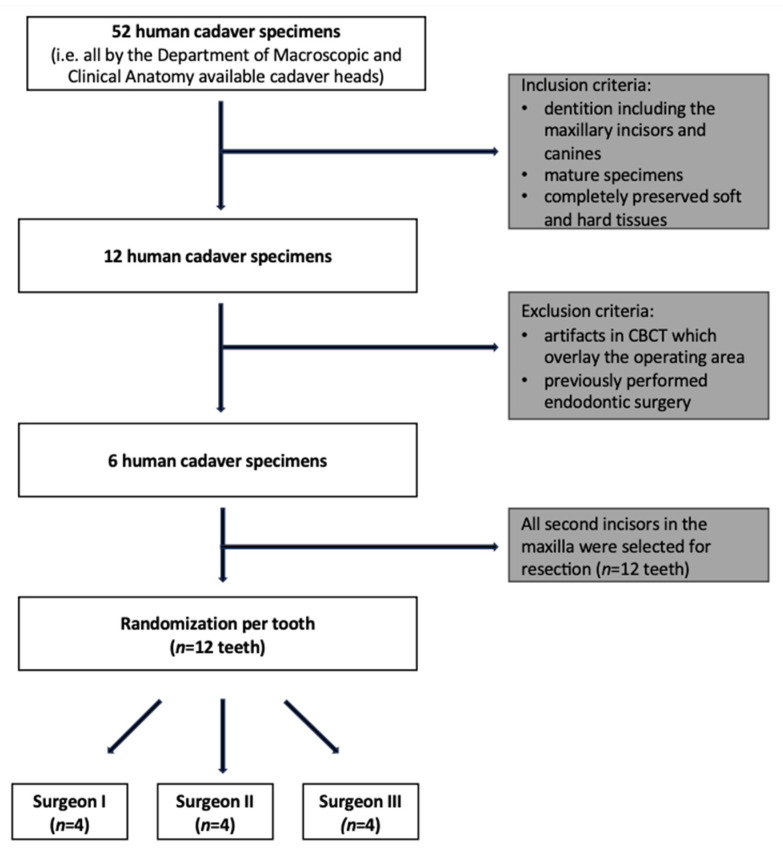
The flowchart illustrates the enrollment and allocation of the study design. The established high-quality preservation method by Thiel was utilized to preserve all human cadaver head specimens [33].

In order to generate an objectively comparable and standardized sample size, this study followed strict inclusion and exclusion criteria. As a first step, all specimens were screened for their eligibility using an intraoral examination performed by an experienced maxillofacial surgeon. An apicoectomy was performed on the right and left maxillary lateral incisors. The primary inclusion criterion for further involvement was anatomical integrity of the maxilla with a dentition including all maxillary incisors and canines. Further inclusion criteria were maturity of the specimens (i.e., adult age) and complete preservation of soft and hard tissues. In the following step, all included specimens underwent a standard dose CBCT scan (96 kV, 5.6 mA, exposure time 9.335 s, field of view (FOV) 23 × 27.5 mm, voxel size 0.400 mm, slice thickness 1 mm). Succeeding the CBCT scan, the application of pre-defined exclusion criteria (i.e., dental restoration material leading to artifacts in the CBCT scan causing significant overlay with the surgical field and a previously performed apicoectomy on the maxillary lateral incisors) resulted in the exclusion of further specimens from this study, as shown in Figure 1.

### 2.2. Augmented Reality System

An AR image guidance system based on the second version of HL (i.e., HL 2) was implemented. The HL 2 features a self-localization algorithm, which maps the surroundings of the user and localizes the device within. This enables users to position virtual content at specific locations within the mapped environment. Users can effortlessly explore said virtual objects by adjusting their viewpoint, resulting in an intuitive and immersive experience. Furthermore, the HL 2 is equipped with a time-of-flight (ToF) depth sensor, capable of capturing an exact 3D representation of the patient in the physical space. The distinct nature of human faces and the relative rigidity of facial structures can be exploited to automatically match the 3D representation of the patient captured by the HL 2 with a 3D model obtained from pre-interventional CBCT imaging. Once registered, imaging data are displayed automatically, overlaid onto the patient. The application is then controlled with a virtual user interface to alternate between different visualization modes, including 3D surface structures and planar slices. All structures are individually displayable, and their transparency and brightness can be adjusted using sliders. In the following sections, the AR system is explained in further detail.

#### 2.2.1. Registration

Patient registration is fully automated in the system used, eliminating external navigation systems, markers, or arduous assembly procedures [31,32]. In short, it uses video frames from the HL 2 video camera, as well as depth maps from the ToF depth sensor. Using a deep learning-based, single-shot, multi-box detector [35], a bounding box around the patient’s face is recognized reliably and in real time. This bounding box is mapped to the depth frame, subsequently reconstructing a point cloud representation of the patient’s face with the application of inverse perspective transformation. The point cloud is matched to the 3D model of the patient’s skin surface obtained from pre-interventional CBCT imaging using a two-stage point-based registration algorithm. First, fast global registration [36] is used to coarsely align the two-point clouds, and this initial alignment is refined using an iterative closest point approach [37]. Then, this algorithm outputs the desired position and orientation of virtual content to ensure that it aligns accurately with the actual anatomy of the patient. A detailed description of the algorithm is discussed in the article by Gsaxner et al. [32].

#### 2.2.2. User Interface

The AR system is controlled entirely with a virtual user interface (UI). Post-patient selection, fully automatic registration is initiated by examining the patient. The position and orientation of virtual content are continuously updated to account for patient movement, as well as to achieve a more precise alignment. However, in cases where a satisfactory alignment has been achieved and the patient is not expected to move (e.g., during sedation), the position and orientation of the virtual content can be established. Further manual refinement of the automatic registration is possible, which may be desirable to account for perceptual misalignments due to the individual anatomy of the user or to account for soft tissue deformations between the pre-operative CBCT scan and the intervention. The UI enables the user to switch between different registration modes, select different anatomical structures to visualize, and adjust various parameters related to content positioning and visualization. The UI is conveniently locked to the users’ left hand, enabling them to bring it up anywhere simply by looking at their flat hand. Closing of the left hand locks the UI to its current position in the room.

#### 2.2.3. Medical Data Visualization

**Figure 2:**
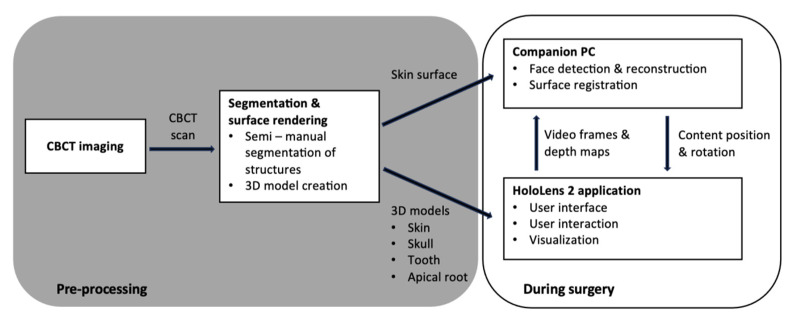
The flowchart demonstrates the digital workflow used in this study.

HL 2 supports several means for adapting the visualization. Anatomical structures can be switched on and off, and the user can interactively modify several parameters to adapt the visualization to their individual preferences. These parameters include options to modify brightness, transparency, and other relevant visual settings. The complete digital workflow is illustrated in Figure 2.

### 2.3. Study Procedure

#### 2.3.1. Randomization

Following the selection of human cadaver head specimens, the allocation of each tooth (maxillary lateral incisor) to one of three experienced surgeons was performed using a digital randomization software (Version 2.1.0, Institute for Medical Informatics, Statistics and Documentation, Medical University of Graz, Graz, Austria; Randomizer for clinical trials: www.randomizer.at accessed on 27 June 2023) by an individual not involved in treatment or evaluation.

#### 2.3.2. Surgery

**Figure 3:**
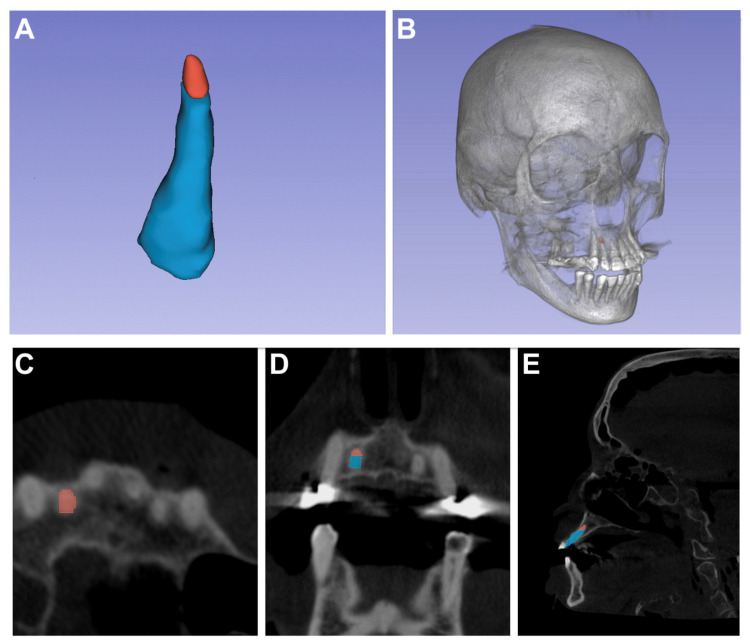
Preoperative rendering of the right maxillary lateral incisor using 3D-Slicer^®^ software. (**A**): A three-dimensional model of the tooth, with the apical 3 mm of the root tip individually color-coded (red). (**B**): A model of the skull with highlighted red root tip (3 mm of the apical root tip of the lateral right incisor). (**C**): An axial plane of the cone beam computed tomography (CBCT) scan presenting the apical root tip in red. (**D**): A frontal view of the CBCT scan displaying both the root tip (red) and the root (blue). (**E**): The left maxillary lateral incisor marked in a sagittal CBCT plane.

Prior to surgical intervention, the entire tooth and the apical 3 mm of the root were marked separately and manually using data from the preoperative CBCT scan as well as 3D-Slicer^®^ software (Version 5.4.0, Slicer Community, USA; Available at: https://www.slicer.org accessed on 27 June 2023 [38]) (Figure 3). Furthermore, the entire facial skeleton and skin surface were additionally segmented for a postliminary visualization in AR using automatic (i.e., thresholding) segmentation and manual refinement. 

Preceding the trial, three experienced surgeons were given a general introduction to the HL 2 and the implemented application of virtual UI. The surgeons were given the opportunity to explore different anatomical structures and visualization modes for training purposes. Several of these training sessions took place well in advance of this study, which allowed the surgeons to become accustomed to the system, gaining both experience and confidence with its application. The outcome of this training was a reduced learning curve, minimized performance bias, and optimized study time during the actual evaluation task. Furthermore, small system-related issues could be eliminated prior to this study.

Before each surgery, the specimen was propped on the operating table, and the instruments for the procedure were prepared. CBCT imaging was registered with the patient using automatic patient registration. Certain cases involving larger soft tissue deformation between the pre-interventional scan and the specimen led to manual adjustment of the registration. Subsequently, the visualized structures and visualization parameters were adjusted for the procedure. The time to complete these preparations was logged as “preparation time”, which also included a recording of the visuals.

**Figure 4:**
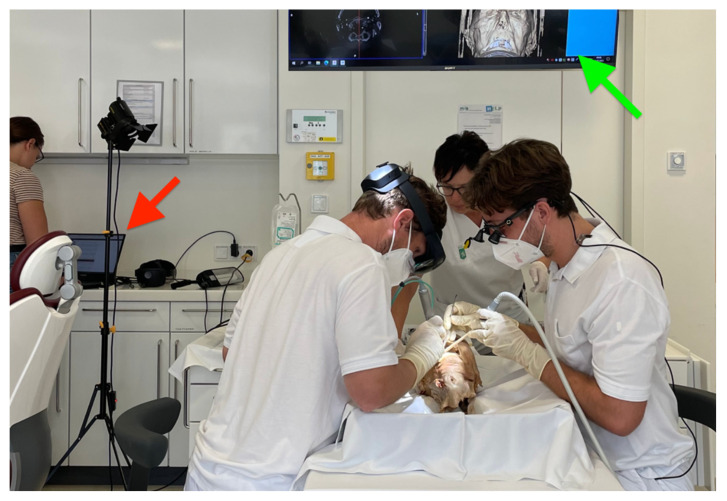
Intraoperative set up at the University Clinic of Dental Medicine and Oral Health, Medical University Graz. The surgeon conducts an apicoectomy on the right maxillary lateral incisor utilizing an augmented reality (AR) image guidance system based on cone beam computed tomography (CBCT) scans. The assistant utilizes illuminated magnifying glasses to optimize lighting during the procedure. A perioperative nurse provides continuous support throughout the surgery. The green arrow indicates the preoperative CBCT scans, while the red arrow marks the technical working station providing the surgeon’s point-of-view. By using the HoloLense 2 as an AR image guidance system, the surgeon’s focus remains completely in the operating field. With this guidance system, the need for the surgeon to alternate attention between the computer workstation and the surgical field is eliminated, allowing complete focus on the operating field by superimposing all image data (including image-based segmentation) into the field of view.

All surgical procedures were performed following a standardized protocol at the University Clinic of Dental Medicine and Oral Health, Medical University Graz. A vertical releasing incision distally to the ipsilateral maxillary canine and a sulcular incision extending to the ipsilateral maxillary central incisor were performed with a #15 blade to create a triangular flap [39]. A full-thickness flap was raised using a Freer elevator and retracted with a surgical standard retractor. The HL 2 guidance system marked the exact spot to create a micro-invasive osteotomy using different sizes of rose head burrs to achieve exposure of the apical tip of the root. Next, a Lindemann burr was used to resect the apical 3 mm of the root tip. Thereafter, the resection surface was smoothened, and the operating field was rinsed using a physiological saline solution (i.e., 0.9% sodium chloride). Retrograde preparation and filling of the root canal were not executed. The mucoperiosteal flap was reattached with 5.0 monofilament non-absorbable single-button sutures. Figure 4 demonstrates the intraoperative setup.

#### 2.3.3. Measurements

For each tooth, the preparation and operating time were documented. The operating time itself was further divided into the time from incision to osteotomy and wound closure time. An accurately calibrated stopwatch was used for time measurements. Preparation time was measured in seconds, while the operating time was rounded up to minutes. These times were compared to 10 apicoectomies that the same experienced surgeons conducted prior to their AR-assisted surgeries. In order to evaluate the usability of the AR guidance system based on CBCT scans in apicoectomies, the well-established System Usability Scale (SUS) was used. The SUS is a validated, standardized questionnaire that classifies the ease of use for a wide variety of products and services such as applications or hardware. This measuring tool consists of ten items on a 5-point Likert scale [40]. Although the scores may range from 0 to 100, these values are not expressed as percentages and should be solely interpreted based on their respective percentile ranking [41].

#### 2.3.4. Statistical Analysis

Descriptive and analytical statistics were used to analyze the gathered data, which were presented as either mean ± standard deviation (SD) or median and interquartile range (IQR). Analytical statistics included the Shapiro–Wilk test, to test the collected data for normality, followed by an ANOVA, and the Kruskal–Wallis-H test, to analyze whether there were statistical differences between users regarding setup time, operation time, and SUS score. For all calculations, a *p*-value of <0.05 was considered statistically significant. All statistical analyses were performed using the statistical Python package pingouin. To additionally visualize this study’s data, tables and boxplots were utilized. All specimens were anonymized before their use with the Division of Macroscopic and Clinical Anatomy at the Medical University of Graz. The recorded data were collected in a case report form. The collection, transfer, and storage of the human cadaver specimens’ image data within this study was carried out in accordance with legal regulations.

## 3. Results

The final sample consisted of 12 teeth (*n* = 12) from six human cadaver head specimens (four male and two female). Each specimen underwent two apicoectomies in the maxilla, one on the left and one on the right side (24 teeth). All teeth showed no apical radiolucency in the preoperative CBCT scans. Each of the three experienced surgeons resected four maxillary lateral incisors, which had at least one adjacent tooth on both the mesial and the distal aspect to imitate structures of interest. No damage to structures of interest (e.g., adjacent roots, maxillary sinus, nasal cavity) during the procedure was observed. Throughout this study, the application encountered a singular instance of failure resulting from overheating, thus necessitating the interruption of this study until the issue was resolved using a power cycle. This incident incurred a downtime of approximately 5 min.

### 3.1. Times

**Figure 5:**
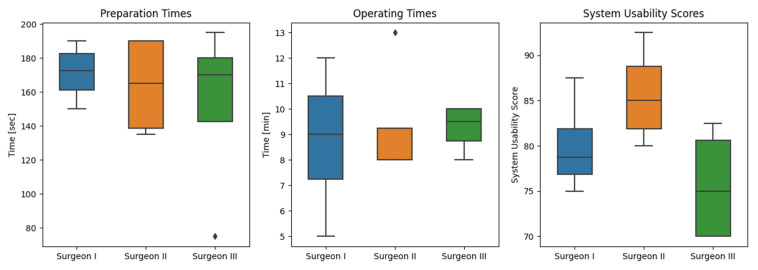
Box plots presenting the differences among the three surgeons regarding preparation time, operating time, and System Usability Scale score. ♦ = extreme outlier.

In total, the preparation times ranged from 75 to 195 s, with an average of 162 s ± 34 s. The preparation time included automatic patient registration as well as manual adjustments (if necessary). The mean operating time was 9 ± 2 min from the first incision to the final wound closure. The fastest operating time recorded was 5 min, while the longest duration required for an apicoectomy was 13 min. Differences regarding the mean operating times among the three surgeons were not statistically significant (*p* = 0.940; Figure 5). Nevertheless, it is worth noting that as the surgeons gained experience with the HL, each of them demonstrated improvement in their respective operating times. The average wound closure time was 3 min 10 s ± 34 s. There was no significant difference detected among the surgeons (*p* = 0.681).

### 3.2. System Usability

The usability of the AR-guided apicoectomy based on CBCT scans was assessed using the well-established SUS questionnaire. After each surgical procedure, each surgeon promptly completed the questionnaire to assess the performance of the HL 2 based on the specific surgery that had been performed. The three operating surgeons rated the AR application with a mean SUS score of 80.4 ± 6.8, which, according to Brooke’s findings [40], indicates a usability level above average (>68). The results of the used SUS questionnaires are presented as plots in Figure 5; means ± SD and median values are shown in Table 1.

## 4. Discussion

The aim of the current study was to evaluate the intraoperative feasibility and usability of an AR image guidance system based on CBCT scans. Therefore, 24 apicoectomy procedures were performed using the HL 2 by three experienced surgeons. The evaluation of the proposed system was performed with the widely recognized SUS. Operating times were additionally measured and compared.

Apicoectomy is an approved treatment option used to preserve endodontically pretreated teeth and has demonstrated a favorable success rate [5,42]. Above all, the implementation of modern techniques like CBCT scans led to an improved diagnosis, root-end resection with minimal to no bevel, retrograde preparation using ultrasonic retro-tips, and the aid of a dental operating microscope, which have substantially improved the success rate [43,44]. Despite the promising outcome rates of the procedure, it is not exempt from inherent challenges. Protection of critical anatomical structures (e.g., maxillary sinus, mandible nerve) and accurate localization of the apical region of the root are among the most challenging steps [10,45]. Particularly, the presence of anatomical variations in tooth roots can give rise to complexities, leading to prolonged operating times, an elevated risk of inadvertent damage to adjacent structures, and prolonged wound healing due to enlarged osteotomies [46].

In order to bypass these difficulties, a variety of different navigation techniques were introduced in the past. These techniques can be roughly divided into static (SN) and dynamic navigation (DN). SN uses presurgical fabricated templates to provide guidance during the osteotomy and root-end resection. Compared with the free-hand technique, this approach has demonstrated a more precise method for accessing the apical portion of the root [8,9,47]. However, the surgical guides must be planned and fabricated in advance, which incurs notable expenses, and they cannot be modified during the procedure. Moreover, rigid anatomical structures (e.g., teeth) are necessary to stabilize the template within the oral cavity to ensure its secure position and minimize potential mobility. The utilization of a rigid surgical template presents various limitations, including impeded visualization of the surgical site, increased heat generation during osteotomy due to diminished contact of cooling fluids with the drills, and restricted mouth opening [19]. In comparison, DN shares similarities with a positioning system comprising basic equipment such as a stereoscopic camera, a computer platform with a screen, and the corresponding navigation software [10]. DN systems for apicoectomies have also been compared with the free-hand method and have shown superior results regarding accuracy and efficiency [9,48,49,50]. Yet, DN faces limitations. In addition to a complex assembly and a time lag for processing commands, DN forces the surgeon to divide their attention between the patient and the navigation system, while effectively coordinating the manipulation of surgical instruments [10,50].

**Figure 6:**
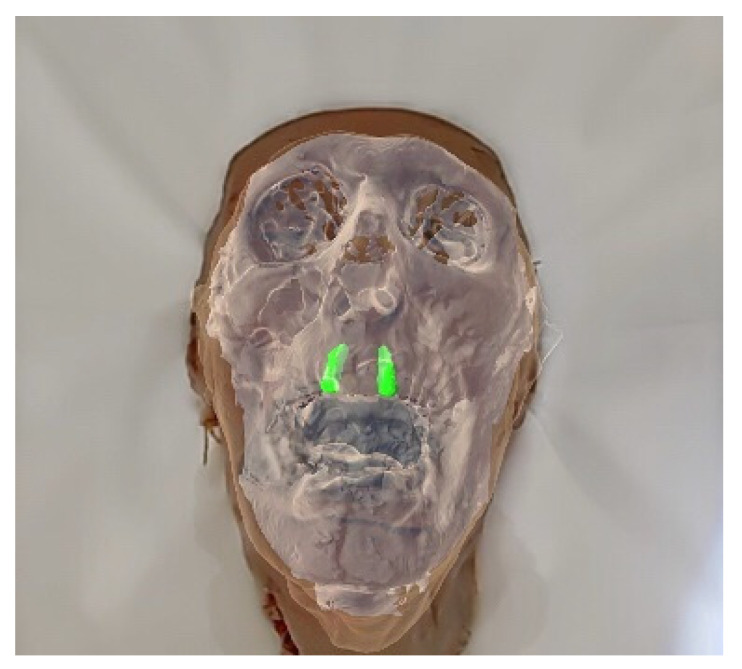
Superimposition of the maxillofacial CBCT scan data over a human cadaver head specimen. Hard tissue and both lateral incisors (in green) are displayed.

With a head-mounted semi-transparent display in front of the users’ eyes, AR can solve this attention deficit with in situ visualization [17]. Preoperative imaging has long been a standard procedure in surgical interventions. Nevertheless, surgeons face shortcomings when using these techniques. They are, for instance, forced to assess 3D imaging on 2D screens and apply this information to the patient in the operating room. The surgeon’s objective is to discern the configuration of anatomical and pathological structures and estimate their size in relation to the surrounding anatomy. AR harbors the potential to seamlessly integrate radiological image data into the prevailing clinical context, superimposing it onto the patient’s anatomy in real time, ensuring correct 3D alignment without any lag [20,31,51,52]. Figure 6 displays the surgeon’s point of view during the surgery.

**Figure 7:**
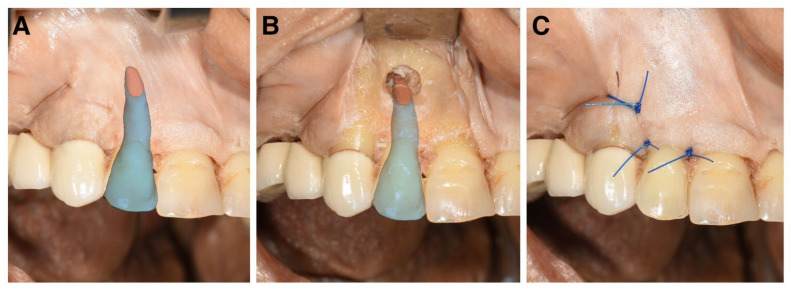
Real-time intraoperative views captured with the HoloLens 2 during the performed apicoectomy on a human cadaver head specimen. (**A**): The image shows a superimposed right maxillary lateral incisor model (blue) with an individually color-coded root tip (red). (**B**): An intraoperative view is shown after incision, osteotomy, and apicoectomy. The apical 3 mm of the root tip was resected according to the preoperatively planned three-dimensional superimposition. (**C**): A postoperative image showing the surgeon’s perspective. The superimposition function on the HoloLens is switched off, showing only the operating field without the superimposed right maxillary lateral incisor model.

According to reviews by Farronato et al. and Joda et al., the predominant focus of AR in the field of dentistry lies within the context of maxillofacial surgical procedures [20,21]. Other investigated domains within dentistry include implantology, oral surgery, dental education, orthodontics, and endodontics. The frequency of studies focusing on maxillofacial surgery can be associated with the scale of the surgical field. AR systems that visualize larger objects tend to find greater applicability in this domain [46]. The most prevalent application of AR systems is in orthognathic surgery, whereby augmented and virtual reality technologies are used for prediction planning and intraoperative navigation [22]. In dental and surgical education, AR has the potential to improve learning outcomes and motor skill acquisition by integrating digital elements into the real world. Learning environments with 24/7 access and the potential objective assessment open up new teaching opportunities [21,22,23]. In implantology, AR mitigates surgeon-induced injuries to critical anatomical structures (e.g., mandible nerve, maxillary sinus, etc.) during the procedure and, in parallel, reduces operating times as well as error rates [18,19,22,24]. According to Chen et al., the implementation of AR in apicoectomies showed enhanced surgical precision regarding bone removal, root-end resection, and bevel angle. However, it was found to increase the duration of surgery when compared with the conventional free-hand technique. The results of the aforementioned study confirm the feasibility of an AR platform for guiding osteotomy procedures and determining apex position in an apicoectomy procedure based on a 3D-printed alveolar model. Critically perused, in this in vitro study, a “screen-through” AR approach was used; hence, the surgeon performed the operation without a direct view of the surgical field [53]. With this technique, the surgeon had to perform the operation solely based on the information gathered from a 2D monitor, which may challenge the accurate visualization of the corresponding anatomical structures, thereby potentially limiting the operating procedure. In contrast, the HL 2 AR system, used in the present study, provides real 3D visualizations of the CBCT data, superimposing anatomical structures and offering a much more comprehensive and intuitive view of the surgical field (Figure 7).

Bosshard et al. conducted a study to assess the accuracy of AR-assisted apicoectomies compared to template-guided apicoectomies using the HL 2 as a see-through head-mounted display. A favorable level of comparability between the two approaches could be shown, indicating that AR performed well in terms of medically relevant trueness and precision. Referencing the author, even a trend toward improved accuracy regarding angular deviation in the resection could be shown. Nevertheless, with respect to the time and effort required, AR assistance was found to necessitate more time subjectively due to the technical preparation involved [54]. However, in that study, the authors used a fixation technique for the pig cadaver mandibles, ensuring a stable position after registration. Additionally, a complete removal of soft tissue was performed, resulting in a simplified experimental setup. In this study, human cadaver head specimens were used to create a life-like situation while operating, which is considered an improvement to the aforementioned study design of Bosshard et al. Additionally, the specimens did not need to be positioned in a pre-determined manner. The registration process relied on an alternative system elaborated upon in the Materials and Methods section. This feature allows shortened set-up time and increased flexibility during the course of the surgery. In the present study, surgical operation times were measured and found to be comparable to in-clinic durations required to perform a conventional free-hand apicoectomy on anterior teeth, typically ranging between 5 and 8 min. For the calculation of these data, 10 apicoectomies performed at the Division of Oral Surgery and Orthodontics up to the cut-off date, the 31st of July, were collected. The mentioned 10 apicoectomies were carried out by the same three experienced surgeons. Additionally, the study team assessed the set-up time needed for technical preparation of the AR system, which was relatively low (averaging at 2 min 42 s) and would therefore not significantly prolong an operating procedure when used in a clinical case. In our user study, the presented system demonstrated above-average usability with an SUS score of 80.4 ± 6.8. Following the SUS rating system introduced by Bangor, Kortum, and Miller, this score corresponds to an “excellent” (6) system on a seven-point scale, ranging from “worst imaginable” (1) to “best imaginable” (7) [41]. Furthermore, a score above 70 indicates that the system is likely to be generally accepted by the target audience. Our study further emphasizes the equivalence in effectiveness between CBCT and the more commonly used CT scans when utilized within an AR framework [55,56]. Hence, our validation supports the potential for seamless integration of CBCT data into an AR workflow, broadening their scope of practical application to the field of dentistry.

While the presented HL 2 prototype application demonstrated its feasibility in the field of dentistry when using CBCT scans for AR-guided dental surgery, it has certain shortcomings that require attention. The current patient registration system lacks support for non-rigid registration; hence, the pre-interventional CBCT scan is assumed to perfectly align with the patient’s exact appearance in situ. While the face is generally a rigid structure, this assumption may not always hold true, especially in situations like swelling, where facial features can be subject to change. In addition, structures in the lower jaw may sometimes not be reliably registered due to its mobility. The position of the mandible may differ from the pre-interventional scan, leading to small inaccurate alignments and tracking of lower jaw structures during the dental procedure. Non-rigid registration could overcome this issue. However, this is a complex process that requires real-time physics-based modeling of soft tissue properties. A further limitation is the current streaming-based approach, which incurs additional latency. The system’s run-time performance becomes heavily reliant on the connection speed and processing capabilities of the companion PC, thus requiring sufficient infrastructure. As a result, users may experience delays or disruptions in the AR application’s functionality. An optimized local processing-based solution, potentially available in the near future, could be a viable alternative to mitigate these concerns. The HL 2 hardware, however, cannot cater to increasing hardware requirements.

Since the adoption of AR systems in dentistry, their potential applications have witnessed exponential growth [57]. Nevertheless, several challenges remain to be addressed before these AR systems can attain widespread commercial use. In the context of surgery, the primary objective of AR systems is to enhance procedural precision. While the software has achieved considerable advancement, the central emphasis should now be directed toward augmenting hardware and technological elements to further optimize AR system performance. In dentistry, the field of operation is often confined to a few millimeters. Under static conditions, and with meticulous calibration, AR overlays exhibit exceptional performance. However, ensuring such optimal scenarios throughout surgical procedures becomes inherently complex. The dynamic nature of a surgeon’s constantly moving head, as well as the patient’s movements, presents significant challenges to the AR system’s seamless functionality. Still, anticipating the availability of increasingly efficient mobile hardware, we optimistically foresee that these challenges can be effectively surmounted with relative ease. The inherent appeal of utilizing AR in oral surgery arises from the predominant focus on bone tissue, which is routinely assessed using CBCT scan analysis, during most surgical interventions. Moreover, the structures necessitating protection, such as the maxillary sinus, adjacent teeth, and mandible nerve, remain stable and resistant to displacement caused by soft tissue manipulation, thus enhancing the compatibility of AR with preoperative CBCT imaging. Apart from the oral and dental surgical field as shown in this study, several other dental procedures may potentially also benefit from the use of AR technology. The implementation of AR technology in these non-surgical dental procedures should be investigated in separate experiments.

## 5. Conclusions

AR-guided apicoectomies using the HL 2 relying on CBCT scans received an excellent rating for system utilization from three experienced surgeons. The integration of AR guidance systems into routine clinical practice is becoming more accessible, especially when relying on diagnostic CBCT scans. High peri-interventional CBCT imaging rates coupled with the presence of hard tissue-bound or well-defined structures requiring protection render this novel technology highly appealing in this specialty. As technology continues to progress, AR systems are poised to revolutionize the field of dentistry, establishing new possibilities for enhanced patient care and treatment procedures.

To further prove the advantages of the AR technology in clinical routine research, the use of a prospective split-mouth design is imperative to compare the AR-guided to the non-guided method.

## Figures and Tables

**Table 1:** Analyzed mean and median values of the applied System Usability Scale are shown. The System Usability Scale was used by the three operating surgeons to assess the performance of the HoloLense 2 in augmented reality-guided apicoectomy procedures.

	Mean (±SD)	Median
Surgeon I	80.00 (±5.54)	78.75
Surgeon II	85.63 (±5.54)	85.00
Surgeon III	75.63 (±6.57)	75.00
Overall	80.42 (±6.81)	80.00

## Data Availability

Not applicable.
